# IVIM Imaging of Paraspinal Muscles Following Moderate and High-Intensity Exercise in Healthy Individuals

**DOI:** 10.3389/fresc.2022.910068

**Published:** 2022-05-31

**Authors:** Erin K. Englund, David B. Berry, John J. Behun, Samuel R. Ward, Lawrence R. Frank, Bahar Shahidi

**Affiliations:** 1 Department of Orthopaedic Surgery, University of California, San Diego, La Jolla, CA, United States,; 2 Department of Radiology, University of Colorado Anschutz Medical Campus, Aurora, CO, United States,; 3 Department of Nanoengineering, University of California, San Diego, La Jolla, CA, United States,; 4 Department of Radiology, University of California, San Diego, La Jolla, CA, United States,; 5 Department of Bioengineering, University of California, San Diego, La Jolla, CA, United States

**Keywords:** intravoxel incoherent motion (IVIM), exercise, paraspinal muscle, MRI, exercise intensity

## Abstract

**Background:**

Quantification of the magnitude and spatial distribution of muscle blood flow changes following exercise may improve our understanding of the effectiveness of various exercise prescriptions. Intravoxel incoherent motion (IVIM) magnetic resonance imaging (MRI) is a technique that quantifies molecular diffusion and microvascular blood flow, and has recently gained momentum as a method to evaluate a muscle’s response to exercise. It has also been shown to predict responses to exercise-based physical therapy in individuals with low back pain. However, no study has evaluated the sensitivity of IVIM-MRI to exercise of varying intensity in humans. Here, we aimed to evaluate IVIM signal changes of the paraspinal muscles in response to moderate and high intensity lumbar extension exercise in healthy individuals.

**Methods:**

IVIM data were collected in 11 healthy volunteers before and immediately after a 3-min bout of moderate and high-intensity resisted lumbar extension. IVIM data were analyzed to determine the average perfusion fraction (f), pseudo-diffusion coefficient (D*), and diffusion coefficient (D) in the bilateral paraspinal muscles. Changes in IVIM parameters were compared between the moderate and high intensity exercise bouts.

**Results:**

Exercise increased all IVIM parameters, regardless of intensity (*p* < 0.003). Moderate intensity exercise resulted in a 11.2, 19.6, and 3.5% increase in f, D* and D, respectively. High intensity exercise led to a similar increase in f (12.2%), but much greater changes in D* (48.6%) and D (7.9%).

**Conclusion:**

IVIM parameter increases suggest that both the moderate and high-intensity exercise conditions elicited measurable changes in blood flow (increased f and D*) and extravascular molecular diffusion rates (increased D), and that there was a dose-dependence of exercise intensity on D* and D.

## INTRODUCTION

Perfusion to skeletal muscle is required to facilitate oxygen and nutrient delivery as well as removal of metabolic waste products. When skeletal muscle undergoes contraction, blood flow to muscle increases substantially, with perfusion increasing from ~3–5 mL/min/100 g at rest (1) to 80 mL/min/100 g or more following exercise (2–5). Mechanisms to increase blood flow involve decreasing vascular resistance through vasodilation of the supplying arteries and arterioles and increasing the vascular recruitment at the level of the capillaries, essentially leading to an expansion of the available capillary surface area for exchange of oxygen between blood and muscle (6, 7).

Non-invasive measurement of whole muscle blood flow both at rest and in response to exercise using magnetic resonance imaging (MRI) is of great interest. Intra-Voxel Incoherent Motion (IVIM) is an MRI technique sensitive to perfusion (8) that has gained interest as a strategy to evaluate blood flow in skeletal muscle (9). IVIM uses diffusion-weighted MR images over a wide range of diffusion sensitivities or b-values, sensitizing the images not only to diffusion but also microvascular blood flow (so-called pseudo-diffusion) (10). The measured data are then fit to a bi-exponential model that describes the contribution of motion in the microvasculature (described by the pseudo-diffusion coefficient, D*) and extravascular spaces (described by the molecular diffusion coefficient, D), weighted by the intravascular signal fraction (f) to the signal decay. Thus, these coefficients provide insight into the relative microvascular blood volume (f) as well as the net displacement of MR-visible water molecules located in the intravascular (D*) and intracellular (D) spaces within a given voxel of the imaging data.

Thus, MRI can not only provide key information on muscle health and adaptive capacity (11–13), but also spatially sensitive information on regions of muscle that have activation impairments in a way that standard measures of muscle activation (e.g., electromyography) cannot (14–16). Identifying these impairments is essential to optimizing function and performance in both healthy individuals and individuals undergoing exercise-based rehabilitation for pain or injury. The ability to use an MRI technique that identifies activation impairments can provide key information on the physiological mechanisms underlying reduced activation in individuals with both healthy and pathological muscle (e.g., pain, disuse, denervation). This information can be used to inform targeted treatments in a patient-specific manner. For example, recently, Shahidi et al. identified perfusion-based impairments in paraspinal muscles of individuals with a history of low back pain after an acute bout of exercise (17). Further, the magnitude of the blood flow response the acute bout of exercise significantly predicted successful reduction in back-pain related disability with a longer-term exercise-based rehabilitation program targeting the paraspinal muscles of interest. Given that individuals with low back pain have been shown to demonstrate changes in muscle health such as atrophy, fibrosis, and fatty infiltration, this technique may provide useful information on pathologies with similar phenotypes. It may also allow clinicians to identify patients who are most likely to successful with exercise-based physical therapy and provides a physiological basis for future research on potential alternative management strategies for those who do not demonstrate the expected response.

Prior studies have demonstrated the ability of IVIM to detect blood flow-related changes in muscle following exercise in muscles of the upper (18–21) and lower extremities (22–25), and back (26, 27). Each of these studies measured the IVIM response to a single, high-intensity exercise stimulus, likely eliciting a maximal hyperemic response. An evaluation of the sensitivity of IVIM to detect blood flow changes to varied exercise intensities in humans has not been performed. Therefore, the purpose of this study was to compare the IVIM results in response to moderate and high intensity exercise to evaluate the ability of IVIM to detect dose-dependent blood flow changes in healthy individuals. We hypothesize that IVIM based measurements of perfusion will be sensitive to paraspinal blood flow changes following moderate and high-intensity lumbar extension exercise in a dose-dependent manner.

## METHODS

### Participants

The University of California San Diego’s Institutional Review Board approved all experimental procedures. Each subject provided informed consent prior to his or her participation, and all study procedures were conducted according to the ethical principles set forth in the Belmont Report. Healthy subjects were recruited from the surrounding community. Potential participants were excluded if they reported a history of low back pain, had prior injuries or surgery to the lumbar spine region, had any contraindication to exercise or to MR imaging (e.g., metal implants, claustrophobia). A subset of the individuals enrolled in the present study also participated in a previously published study (17). Each subject came to the laboratory for two experimental sessions on different days—a minimum of 72 h apart—during which identical MR imaging was obtained before and after either a high or moderate intensity resisted lumbar extension exercise bout.

### Imaging and Exercise Protocol

**FIGURE 1 |:**
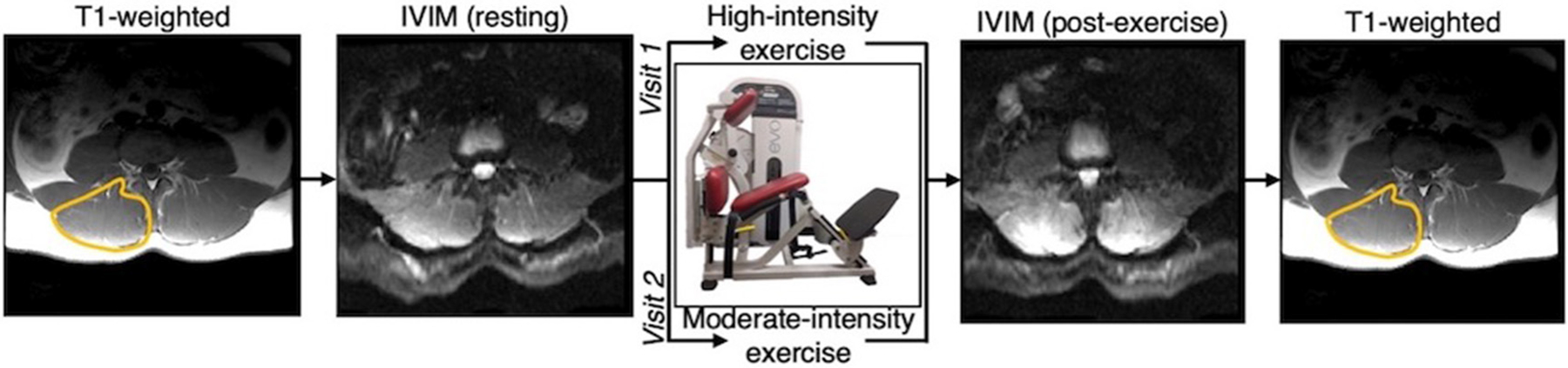
Overview of experimental design. Participants were scanned at rest and following either a moderate or high intensity resisted lumbar extension exercise paradigm. Moderate and high intensity exercise sessions were performed on two separate days. T1-weighted anatomical reference images were acquired before and after the pre- and post-exercise IVIM data, respectively, and used to define the paraspinal muscles bilaterally.

The experimental protocol is illustrated in Figure 1. All imaging was performed on a 3.0T MR imaging system (GE MR 750, GE Healthcare; Waukesha, WI) with a CTL phased-array spine coil for signal reception. Subjects were positioned head-first, supine and centered at ~ the third or fourth lumbar vertebrae (L3/L4). IVIM data were collected at rest (after ~10–15 min in the supine position) and immediately after a bout of moderate or high intensity bout of resisted lumbar extension exercise performed on a lumbar extension machine (Nautilus Evo, Nautilus Inc.) outside the scanner. For the high-intensity exercise, individuals were instructed to target a perceived exertion (RPE) of 6–8 out of 10 on a modified Borg Scale by self-paced exercise with the load set to 60% of their body weight. For moderate-intensity exercise, the targeted RPE was 3–4 out of 10, with the load set to 40% of their body weight. For both intensities, participants were instructed to exercise for ~3 min, or whenever they reached the targeted RPE. The subject immediately returned to the scanner for post-exercise imaging (Figure 1).

**FIGURE 2 |:**
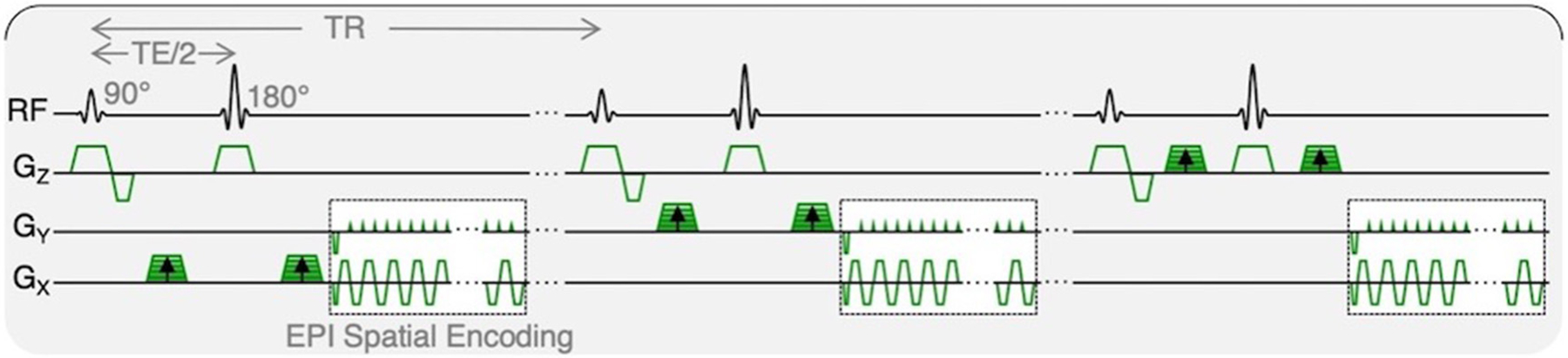
IVIM pulse sequence diagram. Diffusion-weighted MR data were acquired with trace weighting and varying b-values, contributing to varied motion sensitivity. Total acquisition time was 347 s.

Resting and post-exercise IVIM data were identically acquired with an axial, multi-slice, 2D, diffusion-weighted, spin echo EPI with: FOV = 256 × 256 mm^2^, slice thickness = 8 mm, 22 slices covering the entire lumbar spine from L1 to S1, acquisition matrix = 128 × 128, TR/TE = 2,295/52.5 ms, flip angle = 90°, spatial-spectral fat saturation, averages = 4, and 3 directions of diffusion encoding with b-values of 0, 10, 20, 40, 70, 110, 160, 220, 300, 400, 500, 600, and 700 s/mm^2^, yielding a total acquisition time of 347 s. The pulse sequence diagram is shown in Figure 2.

### Image Analysis

As described previously (17), diffusion-weighted images were preprocessed with phase and distortion correction using TOPUP (28, 29) and subsequently denoised using a principal component analysis filter (30). Data were spatially smoothed with a 2D median filter with a 3-voxel radius, and then averaged over the 3 diffusion encoding directions and the 4 temporal repeats for each b-value.

Given the varied strength of the diffusion-weighting gradients, the voxelwise signal intensities as a function of b-value will be sensitive to the underlying microscopic motion. As the scale of motion in the microvasculature vs. intracellular spaces differs substantially, a bi-exponential model can be used that describes both the contribution of motion in the microvasculature (described by the pseudo-diffusion coefficient, D*) and extravascular spaces (described by the molecular diffusion coefficient, D) to the signal decay as:

(1) $$\text{S}(\text{b})/{\text{S}}_{0}=(1-\text{f}){\text{e}}^{-\text{bD}}+\text{f}\;{\text{e}}^{-\text{bD}*}$$

where S(b)/S_0_ is the measured signal at each b-value, normalized by the non-diffusion weighted acquisition, D the molecular diffusion coefficient, D* the pseudo-diffusion coefficient, and f is the perfusion fraction, representing the fractional volume of blood in each voxel.

**FIGURE 3 |:**
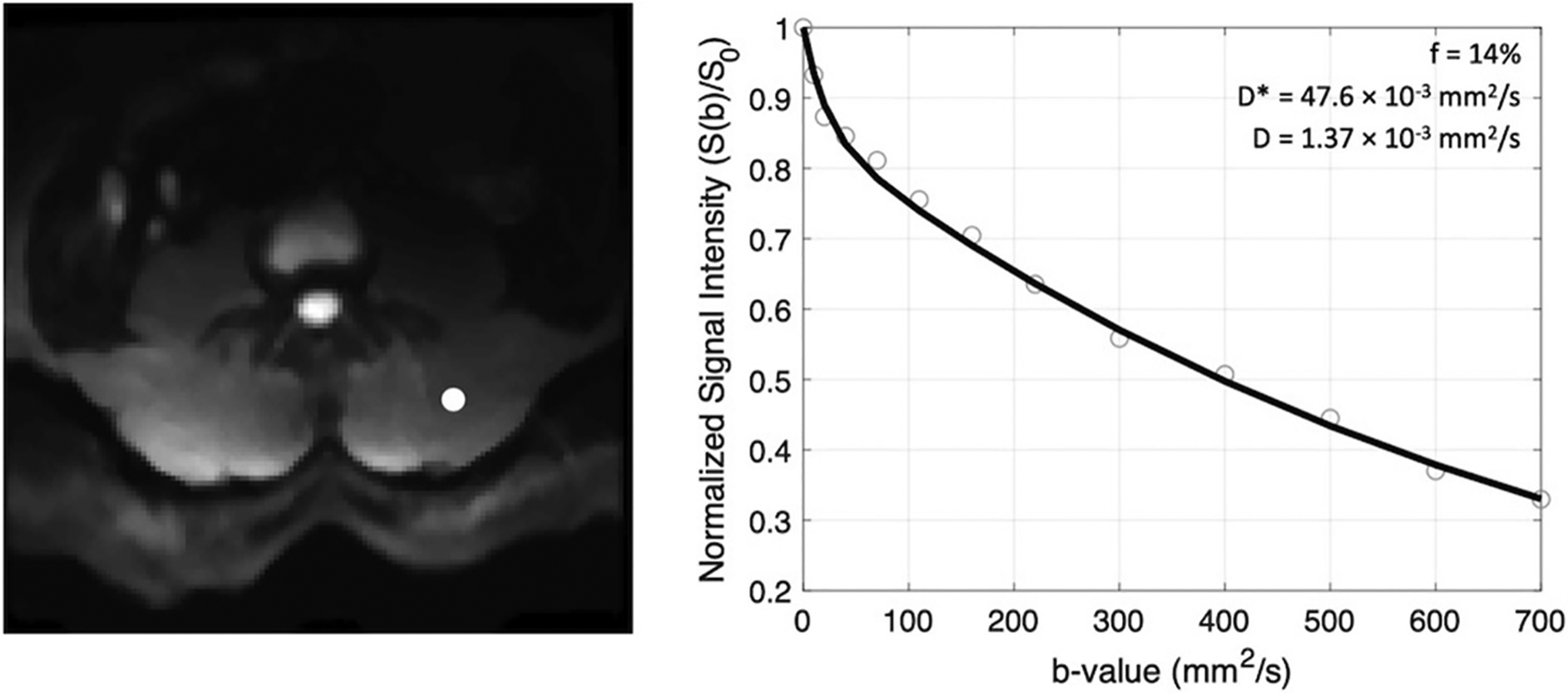
Example of signal decay as a function of b-value (right) shown for the voxel indicated by the white circle on the left panel. The normalized signal intensity data are fit to a bi-exponential function and coefficients f, D*, and D are recorded.

The scale of mean squared displacement of water molecules in the intra- vs. extravascular spaces differs by ~ an order of magnitude. Extravascular diffusion is governed by Brownian motion, whereas the displacement in the intravascular space is related to both Brownian motion and the convective flow of blood in the capillaries (10). Separation of the diffusion and pseudo-diffusion coefficients is therefore possible because contribution from intravascular motion (i.e., D*) to the overall signal decay at high b-values is negligible, meaning that the signal decay at high b-values is governed only by molecular diffusion, D. Here, a three-step constrained fitting approach was used to solve for parameter maps of the coefficients D, f, and D* from the slice-wise IVIM data (Figure 3). First data obtained at b >200 s/mm^2^ (i.e., the high b-value regime) were log-transformed and fit to a first-order polynomial as: $\text{ln}(S(b>200))=-bD+S_{0}^{\prime }$ in order to obtain D. S_0_’, the y-intercept, was then used to calculate the perfusion fraction as: $f=1-S_{0}^{\prime }/S_{0}$. Once f and D were determined from these first two steps, data from all b-values were used to solve (eq. 1) for D* using a non-linear least-squares fitting approach (31). Boundary conditions were applied for all parameters using the following constraints: 0–0.5 for f, 1.5–500 × 10^−3^ mm^2^/s for D*, and an upper bound of 2.5 × 10^−3^ mm^2^/s (the coefficient of free diffusion of water at body temperature) for D. In addition, from the resulting parametric maps, the product of f and D* (fD*) was computed, a parameter most closely related to microvascular blood flow (32).

T1-weighted anatomical reference images were obtained prior to the resting IVIM data and following the IVIM images after exercise. These T1-weighted images were acquired with the same FOV, slice thickness, and image orientation as the IVIM data (Figure 1). Regions of interest (ROIs) were defined by a single observer on the T1-weighted images for the bilateral erector spinae and multifidus muscles combined, using previously described definitions of the muscle boundary (33). ROIs were then transferred to the IVIM parameter maps then eroded to avoid voxels that may be impacted by partial volume artifacts. Data within the ROIs were averaged across the lumbar extensor muscles from L1 to S1 to obtain the mean f, D, and D* at rest and following exercise for each participant.

### Statistical Analysis

Two-way within-subjects repeated-measures analysis of variance (RM-ANOVA) tests were used to compare IVIM parameters before and after exercise between the moderate- and high-intensity exercise bouts. *Post-hoc* paired samples tests were used to evaluate significant differences among individual exercise intensities (e.g., resting vs. following moderate intensity exercise). *Post-hoc* tests were corrected for multiple comparisons using the Sidak method. In order to determine parameter estimates reflecting the amount of change in IVIM measures distinguishing high vs. moderate intensity exercise, univariate regressions were performed for the change in parameters from baseline against the exercise state (moderate and high) for significant variables. In all cases, a *p*-value of 0.05 was considered statistically significant. All statistics were performed using SPSS Statistics (Version 21, IBM, Armonk, NY). All data are reported as mean ± standard deviation unless otherwise noted.

## RESULTS

A total of twelve healthy individuals (6 male, age range = 21–63 years old) were recruited to participate in this study. Data from one participant was excluded due to image artifacts. In the remaining 11 participants, all participants were able to achieve the targeted RPE for both moderate (RPE = 3.4 ± 0.9) and high intensity (RPE = 7.1 ± 1.0) exercise (Table 1). By design, there were significant differences of RPE and resistance weight between the moderate and high-intensity exercise bouts, however differences were not observed between exercise duration (*p* = 0.10) or time between the end of exercise and the start of the post-exercise IVIM acquisition (*p* = 0.65).

**FIGURE 4 |:**
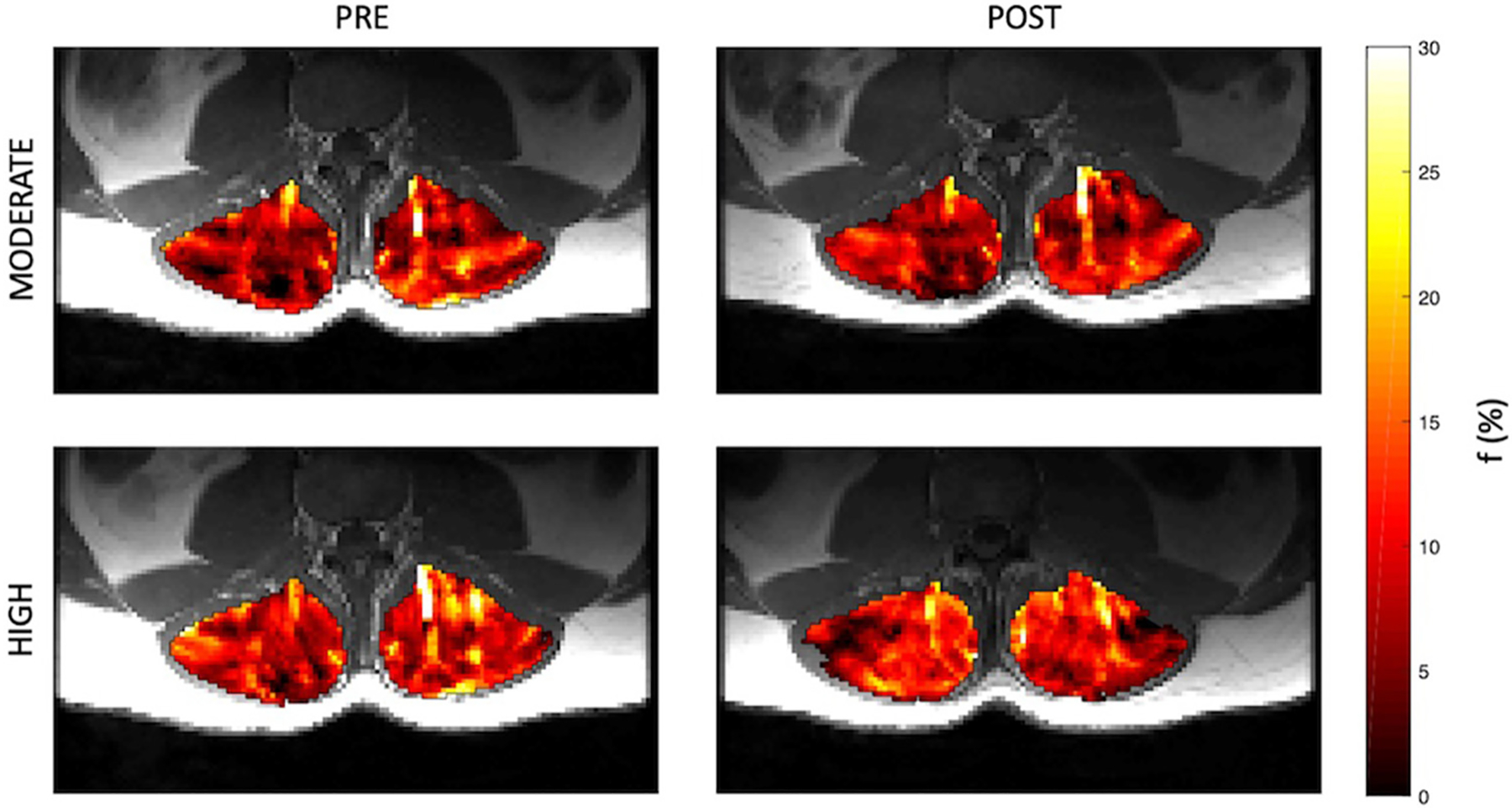
Representative parameter maps of perfusion fraction, f, before and after both moderate (top) and high (bottom) intensity resisted lumbar extension exercise.

**FIGURE 5 |:**
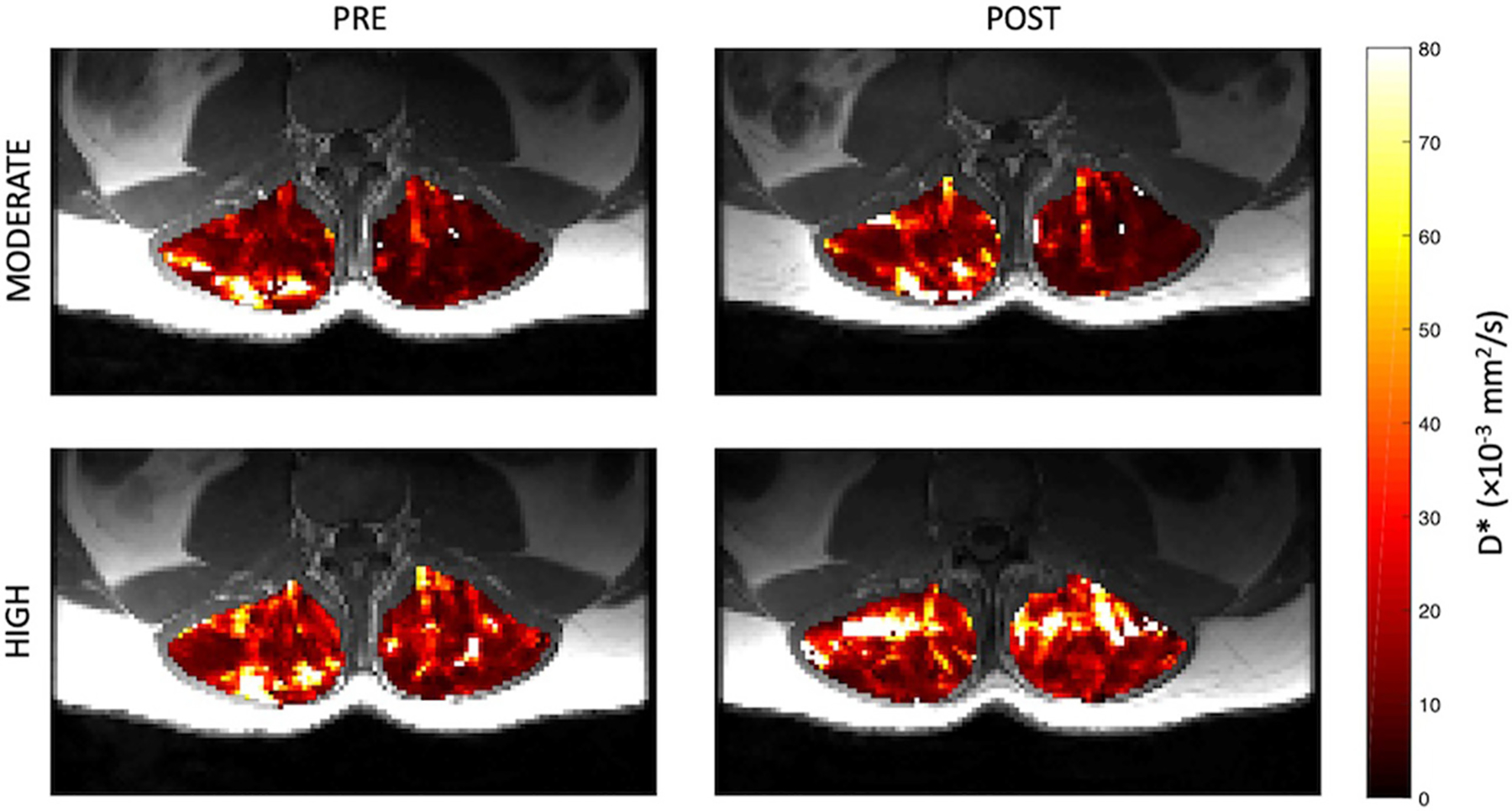
Representative parameter maps of pseudo-diffusion coefficient, D*, before and after both moderate (top) and high (bottom) intensity resisted lumbar extension exercise.

**FIGURE 6 |:**
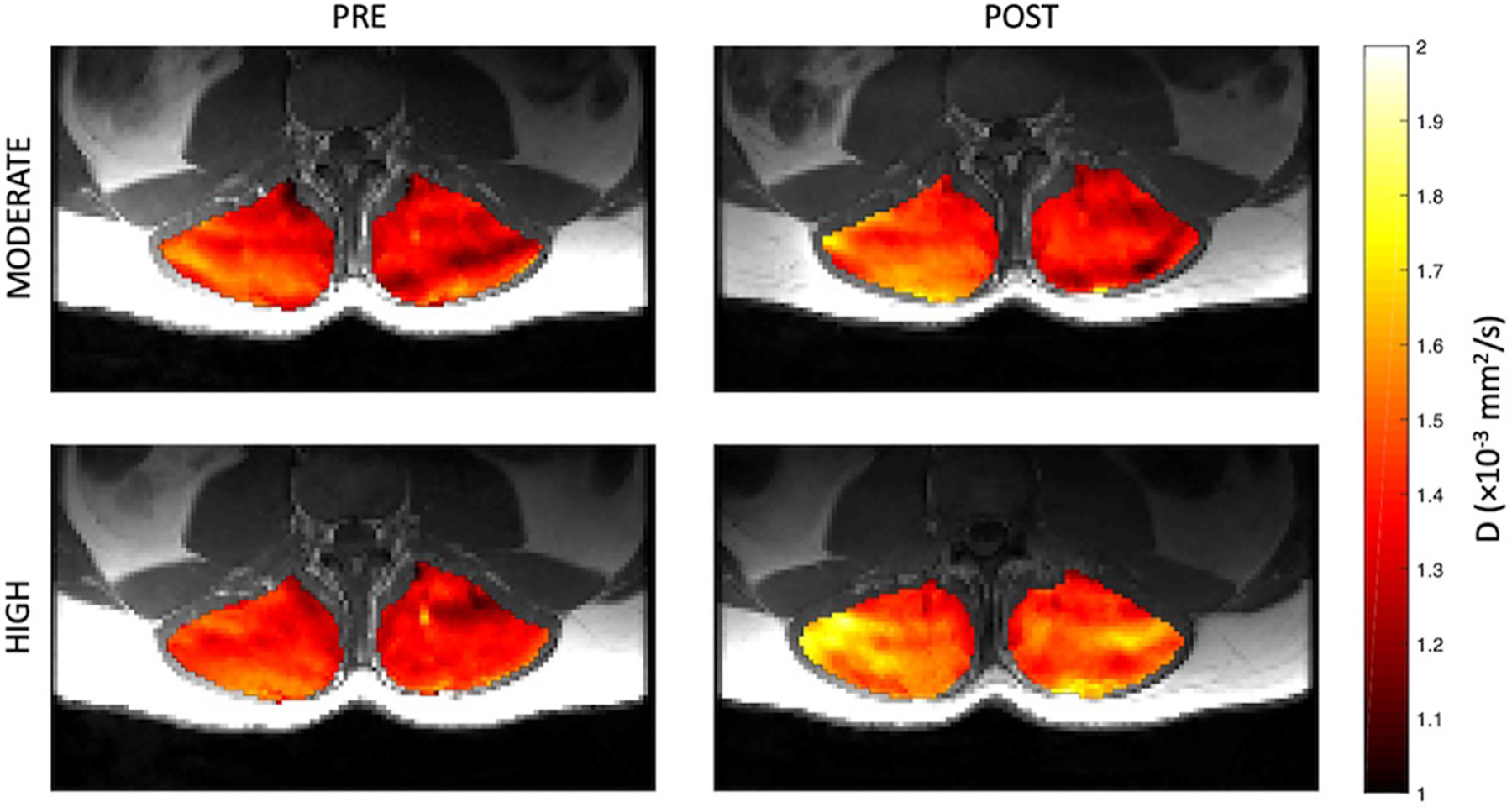
Representative parameter maps of diffusion coefficient, D, before and after both moderate (top) and high (bottom) intensity resisted lumbar extension exercise.

**FIGURE 7 |:**
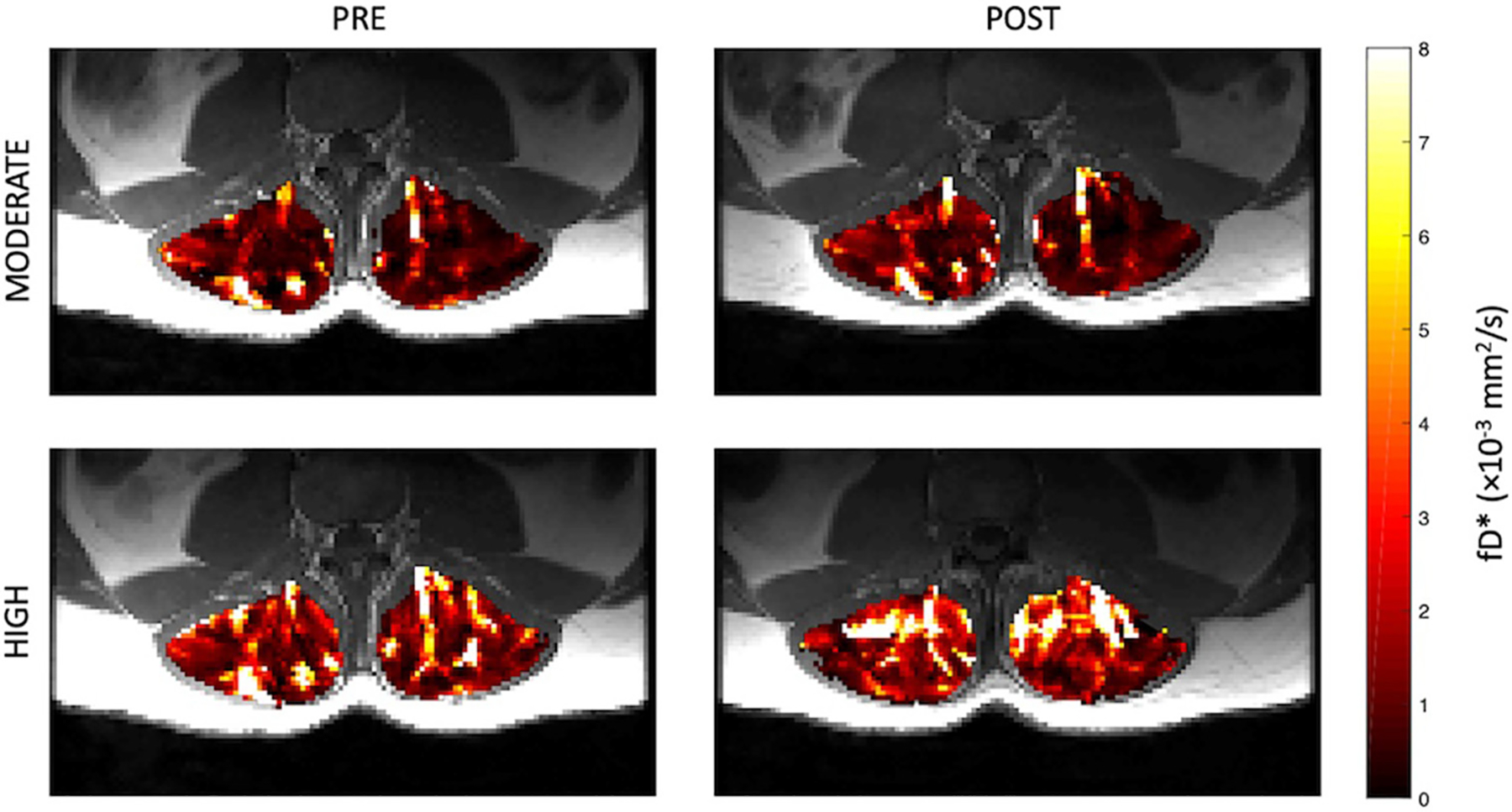
Representative parameter maps of fD* before and after both moderate (top) and high (bottom) intensity resisted lumbar extension exercise.

**FIGURE 8 |:**
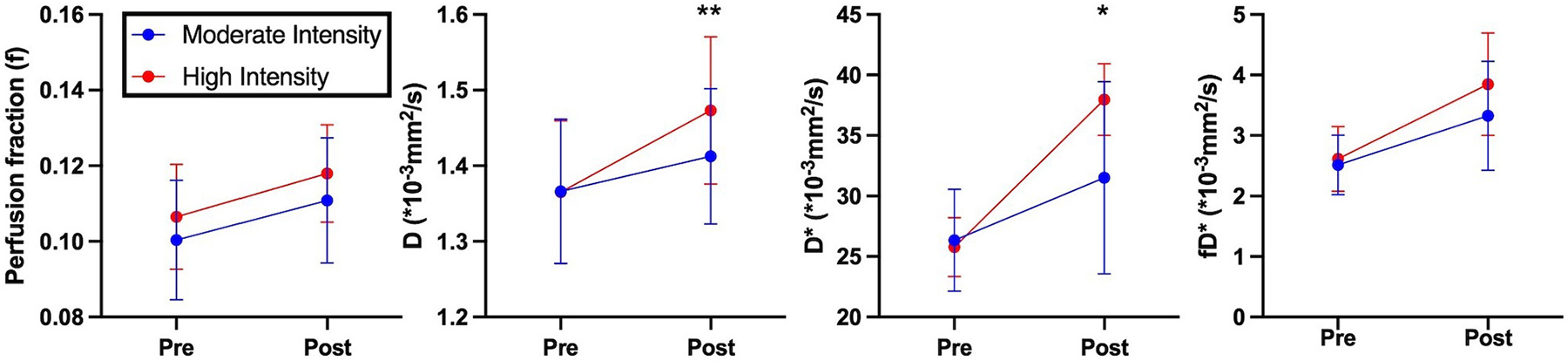
IVIM based perfusion measurements of volunteers pre- and post- exercise. Blue indicates moderate intensity exercise. Red indicates high intensity exercise. Asterisks indicate significant differences in perfusion measurements post exercise between moderate and high intensities (*p < 0.05, **p < 0.01).

Representative parameter maps for each of the IVIM coefficients are shown in Figures 4–7 at rest and following moderate and high-intensity exercise. These images demonstrate the increase of blood flow and molecular diffusion brought about by exercise. The IVIM parameters at rest were not significantly different between visits (f: *p* = 0.14; D*: *p* = 0.71; fD*: *p* = 0.61; D: *p* = 0.93). Overall, both moderate and high intensity exercise elicited an increase in perfusion fraction (*p* = 0.003) and mean squared displacement of water molecules in the intravascular (D*: *p* < 0.0001; fD*: *p* < 0.0001) and extravascular (D: *p* < 0.0001) spaces (Figure 8; Table 2). Moderate intensity exercise led to a 11.2 ± 12.9% increase in f, 19.6 ± 21.7% in D*, 33.0 ± 26.4% in fD*, and 3.5 ± 2.4% in D. High-intensity exercise yielded a 12.2 ± 16.9% increase in f, 48.1 ± 13.9% in D*, 48.6 ± 23.4% in fD*, and 7.9 ± 1.9% in D. The RM-ANOVA revealed a significant interaction between time (e.g., pre-post exercise) and exercise intensity for both D* (*p* = 0.004) and D (*p* = 0.001), but not f (*p* = 0.88) or fD* (*p* = 0.20). *Post-hoc* tests revealed a significant difference between exercise intensity post exercise for both D (*p* = 0.002) and D* (*p* = 0.015) but not f (*p* = 0.177) or fD* (*p* = 0.144). Univariate regression models revealed that a mean ± SE increase of 4.46 ± 0.91% in D, and an increase of 28.56 ± 7.77% in D* reflects the expected increase in exercise intensity (relative to baseline) between moderate and high (*p* < 0.001, and *p* = 0.001, respectively).

## DISCUSSION

The results of this study demonstrate that IVIM coefficients are stable at rest between days and that these parameters are sensitive to paraspinal microvascular blood flow and molecular diffusion changes in a dose-dependent capacity in response to an acute bout of resisted lumbar extension exercise. In accordance with our hypothesis, we found that high-intensity exercise elicited a larger magnitude of changes in IVIM coefficients D* and D compared to moderate-intensity exercise, despite quite limited differences in RPE between the two exercise bouts. Interestingly, while exercise led to an increase in f and fD*, there was no difference found between the moderate and high-intensity exercise conditions. Prior to this investigation, a direct comparison of the IVIM response to varying exercise intensities in the same human subjects, regardless of muscle of interest had not yet been performed.

The increases in each parameter with exercise observed, are in agreement with prior studies investigating the exercise response of the paraspinal muscles to a single intensity of exercise with IVIM. Hiepe et al. evaluated the change in IVIM parameters ~2 min after an isometric back extension exercise that was perceived as hard, reporting an increase in both D and f (27). Similarly, Federau et al. compared the change in IVIM parameters after dynamic lumbar extension exercise performed until exhaustion in both healthy individuals and patients with adolescent idiopathic scoliosis (26). In healthy individuals, they found a significant increase in all IVIM parameters in response to the high-intensity exercise protocol. In a previous study including a subset of individuals enrolled in the present study (17), the response to high-intensity exercise was compared between people with a history of low back pain and healthy controls. The results of that study illustrated the sensitivity of IVIM to detect clinically significant changes in the blood flow response to exercise between individuals with and without low back pain. A study by Lyu et al. investigated the IVIM response to varying exercise programs and durations at serial timepoints after exercise in Sprague-Dawley rats (34). However, this experimental design may not be sensitive to the acute effects of exercise given that their shortest post-exercise data were collected 30 min after exercise cessation. Nevertheless, significant differences between D and D* were observed between exercise protocols, f was not reported in this study.

The increase in molecular diffusion coefficient in response to exercise has been previously attributed to changes in myofiber diameter or tissue heating. While not directly evaluated with IVIM, prior studies have demonstrated a sensitivity between Blood Oxygen Level Dependent (BOLD) imaging, T2-weighted imaging, or diffusion-weighted imaging with the degree of exercise intensity. For example, T2-weighted imaging has been shown to be directly related to exercise intensity (35) and exercise-related glucose uptake measured via FDG-PET in healthy individuals (36). Along with diffusion imaging, T2-weighted imaging has been shown to be sensitive to increased intra- and extracellular fluid shifts, increased blood flow, and microvascular flow (37, 38). However, Froeling et al. demonstrated that while traditional T2-weighted fat suppressed MR images were unable to detect changes in different muscles after prolonged endurance exercise (marathon running), diffusion tensor imaging (DTI) detected intermuscular diffusivity changes, suggesting that diffusion-based imaging may be more sensitive to exercise-induced fluid shifts (39). Similarly, BOLD imaging is thought to be sensitive to blood oxygenation level changes in response to varying exercise intensities, although some data suggests that BOLD signal is less sensitive at higher exercise intensities (40). Although these alternative imaging methods have been used to evaluate differences in exercise intensity with varying levels of sensitivity, the ability to utilize a technique that can distinguish between healthy and pathological states is also of value. Because IVIM provides information specific to capillary blood flow, it provides unique information that is not available using the aforementioned MRI techniques. Given that impairments in capillary function or density are a common feature of pathological muscle in a variety of conditions including spine pathology, diabetes, peripheral arterial disease, and even across the spectrum of aging, the ability to utilize a technique that is sensitive to the capillary function in the context of an exercise challenge allows for potentially broad applicability in commonly seen patient populations.

Contrary to our hypothesis, we did not observe the same dose-dependent increase in perfusion fraction that was observed for the other parameters. This may be due to the way that the control of blood flow at the local level is mediated by microvascular units, a group of ~2–20 capillaries fed by a single terminal arteriole. Microvascular units represent the smallest functional unit of blood flow regulation. At submaximal exercise intensity, only a fraction of myofibers, dispersed throughout the muscle, are activated, as governed by individual motor units. Given the spatial incongruity between motor units (dispersed throughout the muscle) and microvascular units (locally constrained), activation of additional motor units with increasing exercise intensity may not lead to a substantial change in the fraction of blood in the capillaries. This relationship between the spatial organization of blood flow control vs. myofiber activation is one potential explanation for the lack of effect of exercise intensity on f. Increasing exercise intensity would, however, cause additional vasodilation of the feeding arteries and resistance arterioles, leading to faster transit of blood throughout the capillaries, hence an increase in D*. Physiologic modeling and phantom experiments could help to elucidate the relationship between changes in blood flow velocity, blood volume, and molecular diffusion on the measured IVIM signal. In addition, the inclusion of a measurement of blood oxygenation at the tissue level would help to fully understand the relationship between the metabolic demand of exercise and the microvascular and musculoskeletal responses.

Our study is not without limitations. While results demonstrated an effect of exercise intensity on the IVIM coefficients D and D*, only two exercise intensities were evaluated. Additional time points following exercise cessation as well as inclusion of more steps of exercise intensity would help to fully define the relationships that were observed. Some prior literature evaluating other imaging techniques to measure exercise response has included concurrent physiologic measures representing muscle activation, including near infrared spectroscopy (NIRS) for blood oxygenation, pH measures, and electromyography (41, 42). Follow-up studies employing multiparametric imaging and complementary physiologic measures would help to better understand the methodologic responses and physiologic underpinning of the observed IVIM signal changes. Additionally, although our group has demonstrated in a prior study that there are differences in IVIM-based exercise responses between healthy individuals and those with low back pain, we did not evaluate how pain, disease, or aging may differentially impact these responses.

## CONCLUSION

Our study is the first to evaluate the effect of varying exercise intensities on muscle perfusion and diffusion using IVIM MRI in healthy human paraspinal muscle. We found that high-intensity resistance exercise of the lumbar paraspinal muscles elicited a larger magnitude of changes in IVIM coefficients D* and D, but not f or fD* as compared to moderate-intensity exercise. Further studies are needed to validate these findings against physiological standards.

## Figures and Tables

**TABLE 1 |:** Results of exercise protocols for moderate and high intensity exercise paradigms.

	Moderate intensity exercise	High intensity exercise
Relative perceived exertion	3.4 (0.9)	7.1 (1.0)
Resistance weight (kg)	31.3 (7.2)	47.6 (9.2)
Exercise duration (s)	161 (34)	175 (20)
Time to start of IVIM acquisition (s)	182 (74)	164 (40)

All data shown as mean (standard deviation).

**TABLE 2 |:** Results of IVIM coefficients at rest and in response to moderate and high intensity exercise.

	Moderate intensity exercise			High intensity exercise		
	*Resting*	*Post-exercise*	*P-value*	*Resting*	*Post-exercise*	*P-value*
f (%)	10.0 (1.6)	11.1 (1.7)	0.018	10.6 (1.4)	11.8 (1.3)	0.037
D* (×10^−3^ mm^2^/s)	26.3 (4.2)	31.5 (7.9)	<0.001	25.8 (2.4)	38.0 (3.0)	<0.001
D (×10^−3^ mm^2^/s)	1.37 (0.10)	1.31 (0.09)	0.020	1.37 (0.09)	1.47 (0.10)	<0.001
fD* (×10^−3^ mm^2^/s)	2.52 (0.49)	3.33 (0.90)	0.004	2.62 (0.53)	3.85 (0.84)	<0.001

All data shown as mean (standard deviation).

## Data Availability

The raw data supporting the conclusions of this article will be made available by the authors, without undue reservation.
